# Recent Advances in Electrochemical and Optical Biosensors Designed for Detection of Interleukin 6

**DOI:** 10.3390/s20030646

**Published:** 2020-01-23

**Authors:** Munezza Ata Khan, Mohammad Mujahid

**Affiliations:** 1 School of Chemical and Materials Engineering, National University of Sciences & Technology, H-12, Islamabad 44000, Pakistan; m.mujahid@stcatz.oxon.org; 2 School of Materials Sciences & Engineering, Nanyang Technological University, Nanyang Avenue, Singapore 639798, Singapore; 3 Pak-Austria Fachhochshule, Institute of Applied Sciences & Technology, Khanpur Road, Mang, Haripur 22650, Pakistan

**Keywords:** interleukin 6, biosensor, cancer detection, electrochemical sensor, optical sensor

## Abstract

Interleukin 6 (IL-6), being a major component of homeostasis, immunomodulation, and hematopoiesis, manifests multiple pathological conditions when upregulated in response to viral, microbial, carcinogenic, or autoimmune stimuli. High fidelity immunosensors offer real-time monitoring of IL-6 and facilitate early prognosis of life-threatening diseases. Different approaches to augment robustness and enhance overall performance of biosensors have been demonstrated over the past few years. Electrochemical- and fluorescence-based detection methods with integrated electronics have been subjects of intensive research due to their ability to offer a better signal-to-noise ratio, high specificity, ultra-sensitivity, and wide dynamic range. In this review, the pleiotropic role of IL-6 and its clinical significance is discussed in detail, followed by detection schemes devised so far for their quantitative analysis. A critical review on underlying signal amplification strategies and performance of electrochemical and optical biosensors is presented. In conclusion, we discuss the reliability and feasibility of the proposed detection technologies for commercial applications.

## 1. Introduction

**Figure 1:**
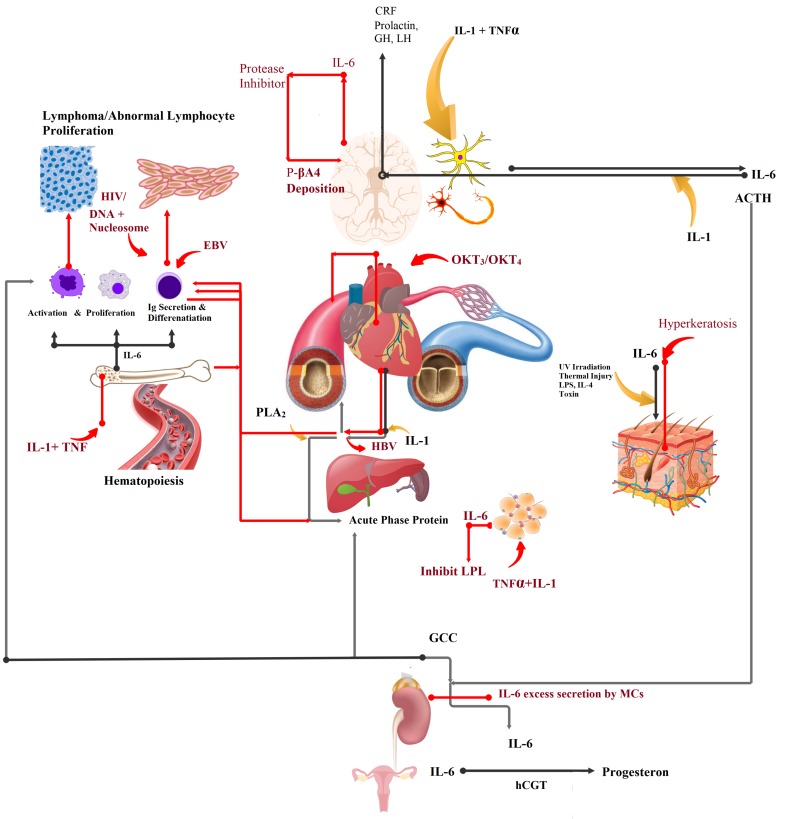
Pleiotropic function of interleukin 6 (IL-6) in humans. Black colored pathway shows normal physiological role, and red-colored pathway indicate impaired functions. CRF = corticotropin-releasing factor; TNF = tumor necrosis factor; LPL = lipoprotein lipase; GCC = glucocorticoid; ACTH = adrenocorticotrophic hormone.

During the past few decades, interleukin 6 (IL-6) has drawn the attention of immunologists and molecular pathologists because of its pleiotropic functions in human body [1,2,3,4,5]. It has key role in immunomodulation, hematopoiesis, and inflammation processes. An over-expression or upregulation of IL-6 can disrupt the normal functions of multiple organ systems in human body, as illustrated in Figure 1. It belongs to the family of glycoproteins and exhibits varying molecular mass, from 26 to 30 kDa depending on cell-specific post-translational modifications [6,7]. There are 212 amino acids in IL-6 [8] which show 65% structural homology with its counterpart produced in mouse/rats [9,10]. IL-6 has a tertiary structure containing four α helices [11], and α helix with terminal -COOH group is involved in receptor binding process. IL-6 is secreted by a variety of cells, like keratinocyte, endothelial cells, neural cells, lymphocytes, and bone cells, when stimulated by specific inducers; however, constitutive expression triggers tumor formation [6]. IL-6 establishes cellular communication by binding with its receptor called interleukin-6 receptor (IL-6R) [6]. It is an integral membrane protein and possesses a conserved region of 90 amino acids belonging to the immunoglobulin supergene family [12,13]. IL-6R structure contains two anti-parallel fibronectin III type domains, each containing seven β folds and between two domains, there is a dip where IL-6 binds to initiate a cascade of cellular reactions [11,14,15]. Once IL-6 fits inside the binding pocket of IL-6R, another 130 kDa protein, called gp130, serves as the signal transducer and facilitates formation of high affinity extracellular receptor binding sites [14,16,17]. The interaction of IL-6 with serum soluble IL-6R is also facilitated by the serum soluble form of gp-130, which also produces negative feedback in serum [18]. It is imperative to understand the clinical significance of IL-6 before we explore the sensing mechanisms developed so far for its quantitative detection because the prognostic values vary with disease type and severity. A careful consideration of this factor will not only help researchers develop strategies that allow detection with higher sensitivity at a wider dynamic range but also extend its application in diagnosis of multiple diseases.

IL-6 invokes immune response through a set of hormones released by the neuroendocrine system following an injury, chronic infection, burn, or internal damage to tissues/cells. Secretion of IL-6 in nerve cells is initiated by IL-1 and the Tumor Necrosis Factor (TNF) [19]. Here, it acts as an autocrine growth factor [20] and synergizes with IL-1 to induce release of hormones, including Adrenocorticotrophic Hormone (ACTH) and corticotropin-releasing factor (CRF) [21,22,23,24,25,26] (Figure 1). The neuroendocrine pathway is positively feed-backed by CRF. In the event of infection, ACTH, in synergy with IL-6, acts on adrenal glands to begin production of the glucocorticoid (GCC) hormone [27,28]. As a consequence, hepatocytes start synthesizing acute phase proteins [29,30], including c-reactive protein (CRP), serum amyloid A, opsonins, and several clotting and healing factors [31,32], under the stimulation of IL-6 and glucocorticoid [30,33]. In higher degree burns, IL-6-induced levels of circulating CRP elevate [34,35], and in sepsis, it increases to 100,000 pg/mL [36] (Table 1). In septic shock and rheumatoid arthritis, Phospholipase enzyme A${}_{2}$ activated by IL-6 shows increased inflammation related activities [37].

Damaged neurons in Alzheimer’s disease (AD) overproduce IL-6 which, in combination with IL-1, activates $\alpha_{2}$ macroglobulin/ $\alpha_{2}$-M [36,38,39,40]. $\alpha_{2}$-M inhibits post-translation modification of the amyloid precursor and triggers deposition of the pathogenic β4 amyloid in the senile plaques. The β4 amyloid is responsible for AD-associated dementia (Figure 1, red-lined pathway). Table 1 shows the abnormally high level of IL-6 in patients with AD.

During hematopoiesis, IL-6 serves as an autocrine growth factor on pluripotent stem cells residing in spleen [52] and bone marrow [53,54], megakaryocytes [55,56,57], myeloid cells [58], plasmacytoma, and myeloma cells [59,60,61,62]. Functional impairment in its growth stimulating activities results in the formation of abnormal mass of tumorous cell in bone marrow [63]. Pathogenic IL-6 inducers that promote abnormal growth and formation of lymphoma in immune cells are Epstein Barr transformed B cell [64,65], OKT${}_{3}$ and OKT${}_{4}$ monoclonal antibodies (in cardiac transplant patients), DNA, and nucleosomes that participate in the manifestation of non-hodgkin lymphoma [66], post-transplant lymphoproliferative disorder (PTLD) [67,68,69], and systemic lupus erythematosus [70,71,72,73], respectively. An abnormally high level of IL-6 in the serum of individuals with plasma cell dyscrasia is associated with advanced multiple myeloma [74]. The prognostic values for the aforementioned disease fall within the range of 5–11,020 pg/mL. IL-6 produced by vascular smooth muscles cells (VSMCs) [75,76] is involved in maintaining heart contractile activity [77]. Its excessive production in VSMCs results in activation of the nitric oxide pathway, which prolongs the vasodilation period. An inadequate blood supply to the heart tissues causes myocardial infarction [78,79,80,81]. Constitutive expression of IL-6 in VSMCs also causes cardiac myxoma [82].

In bones, IL-6 produced by osteoblasts and osteoclasts under the stimulation of TNF, IL-1, or parathyroid hormone serves as osteotropin cytokine and regulates the bone metabolism [83,84,85]. The estrogen level drops down in aging women, which results in increased production of IL-6 by osteoclasts, thereby accelerating the bone resorption process [86,87]. It can be used as a prognostic marker to determine the severity of osteoporosis in patients with Rheumatoid Arthritis [88,89,90]. It is also correlated with an increased level of an inflammatory protein, called CRP, which triggers and assists building up of inflammatory tissue lining in the patient’s joint [91,92].

Neurath et al. [93], in 1992, explained IL-6 as a facilitator of the hepatitis B virus, where it assists it in the progression of hepatocarcinoma (HC). Level of IL-6 in Hepatitis B virus-infected patients can be used as a prognostic tool for cancer susceptibility. Damaged mesangial cells in the kidneys also overproduce IL-6 and cause mesangial proliferative glomerulonephritis [94,95]. Hyperkeratosis in Psoriasis patients is facilitated by IL-6, and it assists in the formation of inflammatory patches/lesions [2,50,96]. Overproduction of TNF/IL-1 increases IL-6 secretion in adipose tissues. Here, it suppresses lipoprotein lipase (LPL) activity which is responsible for fat metabolism. IL-6 levels in serum of cachexia patients can be used to trace LPL activity [97,98,99].

Figure 1 illustrates the pleiotropic role of IL-6 and its clinical significance, whereas Table 1 shows the levels of IL-6 produced in different pathological conditions. The discussed multi-functional role of IL-6 indicates the importance of its quantitative detection in assessing the severity of different pathological conditions. In this review, we will discuss the structure and sensing mechanism of different electrochemical and optical biosensors designed for IL-6 detection in detail and assess its performance in terms of sensitivity, selectivity, dynamic range, and stability.

## 2. Electrochemical Biosensors

### 2.1. Detection Mechanism

**Figure 2:**
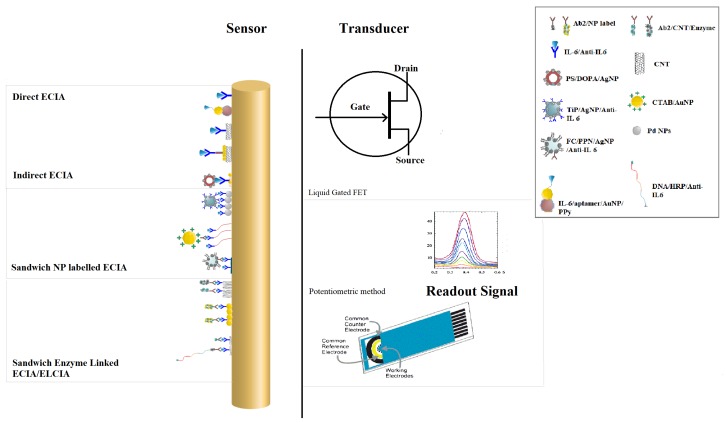
Different sensing mechanisms developed for IL-6 detection. Direct electrochemical immunoassay (ECIA) based on IL-6 and the primary antibody (Ab${}_{1}$) interaction [100]; using a conjugate of polypyrrole-Au Nanoparticles-aptamer [101]; carbon nanotubes (CNTs) coated with (Ab${}_{1}$) [102]; carbon nanotubes coated with gold nanoparticles (AuNPs) and (Ab${}_{1}$) [103]. Indirect electrochemical immunoassay in which a competitive antigen bound to AgNP-coated Polystyrene nanobeads [104]. Sandwiched electrochemical immunoassay in which a secondary antibody (Ab${}_{2}$) is either coated with nanoparticles of Titanium Phosphate (TiP) and silver [105]; gold [106]; ferrocene (FC)-loaded porous polymeric nanoparticle (NP) [107]; or linked with enzymatic label directly or through mutli-enzyme approach using carbon nanotubes [108,109,110].

A sensor’s performance is dependent on its architecture. We will discuss in this review only the biosensors which have been designed for IL-6 detection by exploiting electrochemical nature and light scattering properties of biomolecules in the past ten years. Figure 2 shows different construction scheme of sensing layer for electrochemical detection of IL-6. Field effect transistors and potentiometer are the widely used transducers for conversion of input signal into readable output signal. Biomolecules immobilized on the electrode surface upon interaction with detecting analyte alter charge transfer properties and ultimately affect the readout signal. The change in the readout signal is translated into the corresponding analyte concentration. Based on the construction scheme, we have classified electrochemical sensors into different types and subsequent subsections discuss each type in detail.

#### 2.1.1. Direct Electrochemical Immunoassay (dECIA)

When the readout signal is generated by the direct interaction of detecting analyte with primary antibody which is immobilized on self-assembled monolayer (SAM) coated on electrode surface, we refer it as direct electrochemical immunoassay (dECIA). This is analogous to direct enzyme linked immunoassay (ELISA) approach which produces fluorescence when the antigen and antibody complexes are formed. Antibodies against IL-6 can be directly immobilized on the surface of a functionalized electrode [100] or nano-features can be introduced in the sensing layer [101,102,103] for signal amplification purposes as shown in Figure 2. A robust dECIA method with a wide linear range was demonstrated by Tertis et al. [101], and aptamer against IL-6 was immobilized on polypyrrole nanoparticles (PPy) decorated with gold nanoparticles (AuNPs) of 50 nm diameter. The wide dynamic range is due to the increased electrode surface area when modified with AuNPs. Another label-free approach of direct IL-6 detection was explained by Chen et al. [102] in 2016, and primary antibody against IL-6 was attached to carbon nanotubes. It was used as a liquid gate for field effect transistor (LGFET) with the source and drain made of gold. The formation of antibody–antigen complex changes the drain current which is used to quantify the concentration of IL-6 in the sample. An electrode modified with single-wall carbon nanotubes (SWCNT) and coated with AuNPs was used as impedance-based sensor, and it offers the greatest sensitivity and the lowest limit of detection (Table 2).

#### 2.1.2. Indirect Competitive Electrochemical Immunoassay (icECIA)

A nanolabeled competitor is used in an indirect competitive electrochemical immunoassay (icECIA), which inhibits binding of the target analyte with the receptor. The readout signal is generated and amplified by the interaction of the nanolabeled competitor and receptor. The rationale behind the indirect detection approach is that the readout signal is inversely related to the concentration of the target analyte. Lou et al. [104] (2014) adopted an indirect approach to detect IL-6 through differential pulse voltammetry method. Electrochemically reduced graphene oxide (ERGO) was coated with Au and Pd nanoparticles, whereas the competitor was composed of dopamine coated polystyrene nanobeads which were decorated with silver nanoparticles (AgNPs) prior to antigen immobilization. At higher concentrations of IL-6 in samples, the receptor is fully occupied by target analyte/IL-6, and a decreasing current was observed. It is due to the lower occupancy of the silver-bound IL-6 on the receptor which improves the charge transfer properties of the electrode and ultimately increases the output current.

#### 2.1.3. Sandwich Nanoparticles Labeled Electrochemical Immunoassay (sECIA-NP)

The use of a labeled secondary antibody (Ab${}_{2}$) specific to the target analyte is an alternative approach to improve the limit of detection (LOD) of biosensors designed specifically for cancer detection. The receptor at the electrode has a primary antibody (Ab${}_{1}$) which, upon binding with the target analyte, produces electrochemical signal. The signal is further enhanced upon binding of Ab${}_{2}$. The choice of label for the secondary antibody is dependent on the level of sensitivity required for the intended sensing application. Electroactive nano-labels can be attached to the secondary antibody to enhance the output signal and, ultimately, the sensitivity of proposed sensor scheme. Titanium Phosphate (TiP) hollow spheres of 40 nm shell with efficient loading of AgNPs found its application in ultra-sensitive detection of IL-6. The secondary antibody was immobilized on TiP/AgNPs hybrid nanospheres and used to bind IL-6 and anti IL-6 complex formed at an electrode modified with superparamagnetic Iron oxide nanoparticles [105]. Figure 2 shows the illustration of this scheme, and Table 2 presents the essential properties of sensor. Li and Yang [107], in 2011, used ferrocene (FC) as an electrochemical label and encapsulated it inside porous polyelectrolytes nanospheres (FC-PPN). The secondary antibody was immobilized on the FC-loaded PPN, and the primary antibody was attached to a graphene oxide (GO)-modified electrode. The biochemical interaction produced an ultrasensitive response of the sensor, with a wide linear range, due to the efficient FC loading in PPN and increased surface area of the GO-modified electrode. Gold nanoparticles stabilized with cetyltrimethylammonium bromide (CTAB), when used as nanolabel for Ab${}_{2}$, also offers reasonable sensitivity for IL-6 detection [106].

#### 2.1.4. Sandwich Enzyme Linked Electrochemical Immunoassay (sELECIA)

In the sandwich enzyme linked electrochemical immunoassay (sELECIA), an enzyme is used to label the secondary antibody, and the number of catalyzed substrate determines the concentration of target analyte in the sample. A commonly used enzyme is horseradish peroxidase (HRP), which is occasionally used in combination with streptavidin-biotin. In a conventional fluorescence-based ELISA, there is a limitation on the number of labels you can attach to a secondary antibody, refer to Figure 2. A dual strategy for improvement in the output signal was introduced by Wang et al., wherein they increased the number of catalytic reactions for a single Ab${}_{2}$-Target-Ab${}_{1}$ complex forming event. The number of HRP labels was significantly increased when a carbon nanotube (CNT) decorated with dopamine/AuNP was used for label attachment Figure 2. The electrode was also modified with dopamine and AuNPs before Ab${}_{1}$ immobilization on it [109]. Later, Wang introduced a DNA supersandwich-based ECIA for IL-6 detection with improved sensitivity and linear response [110]. A DNA capture probe was attached with Ab${}_{2}$ through biotin–streptavidin linkage, and then signal probe with HRP label was hybridized with it. The concatenation of the signal probe amplifies the output signal and produces an ultra-sensitive response for IL-6 detection.

A multi-label design was explained by Malhotra et al. [108], and they obtained 60-fold lower LOD by increasing the number of HRP on multiwall carbon nanotube (MWCNT) for a single target binding event. The receptor was also modified with upright SWCNT forest to offer high electrode surface area for the immobilization and subsequent formation of antibody-antigen complexes. A schematic of this sandwich ELECIA is provided in Figure 2.

### 2.2. Electrode Interface

**Figure 3:**
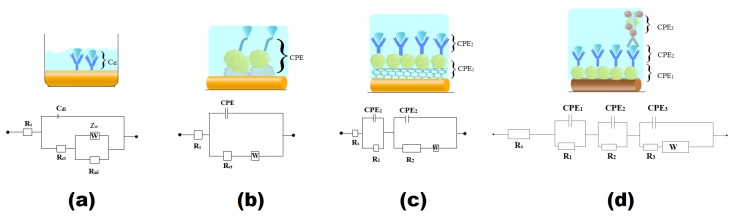
Electrode interface of different ECIA scheme with their equivalent circuits: (**a**) dECIA [100], (**b**) dECIA with nanolabel of Ppy/AuNP and aptamer specific to IL-6 for binding analyte [101], (**c**) dECIA with electrode coated with SWNT (single-walled carbon nanotubes)/AuNP [103], and (**d**) sELECIA with nanolabel of CNT/AuNP and enzyme label Horseradish Peroxidase [109].

In this section, we will explain the electrode interface of the previously mentioned sensors’ structure, including dECIA, dECIA with nanolabels, and sandwich ECIA with nanoparticles and an enzyme label. Figure 3 represents the equivalent circuits of the respective examples. The bio-recognition layer coated at the surface of metal electrode acts as double layer capacitor and difference in charge at interface produces the sensor’s response [112]. The biological macromolecules at the surface compensate for Donnan potential and put limitations on the field effect transistor (FET)-based electrochemical sensing approach [113,114]. The detecting potential decays when the thickness of the biorecognition layer extends beyond 1 nm [115]. Advancements in nanofabrication techniques have made it possible to scale down the electrode geometry to Debye length, and the charge compensation effect can be considerably reduced [116,117,118,119,120,121,122]. Chen et al. [102], in 2016, proposed a liquid-gated FET sensor for IL-6 detection and, based on the aforementioned facts, they significantly reduced the limit of detection and improved the dynamic range (Table 2). The electrode interface of the FET-based design has been omitted because it has already been thoroughly discussed by [123].

**Figure 4:**
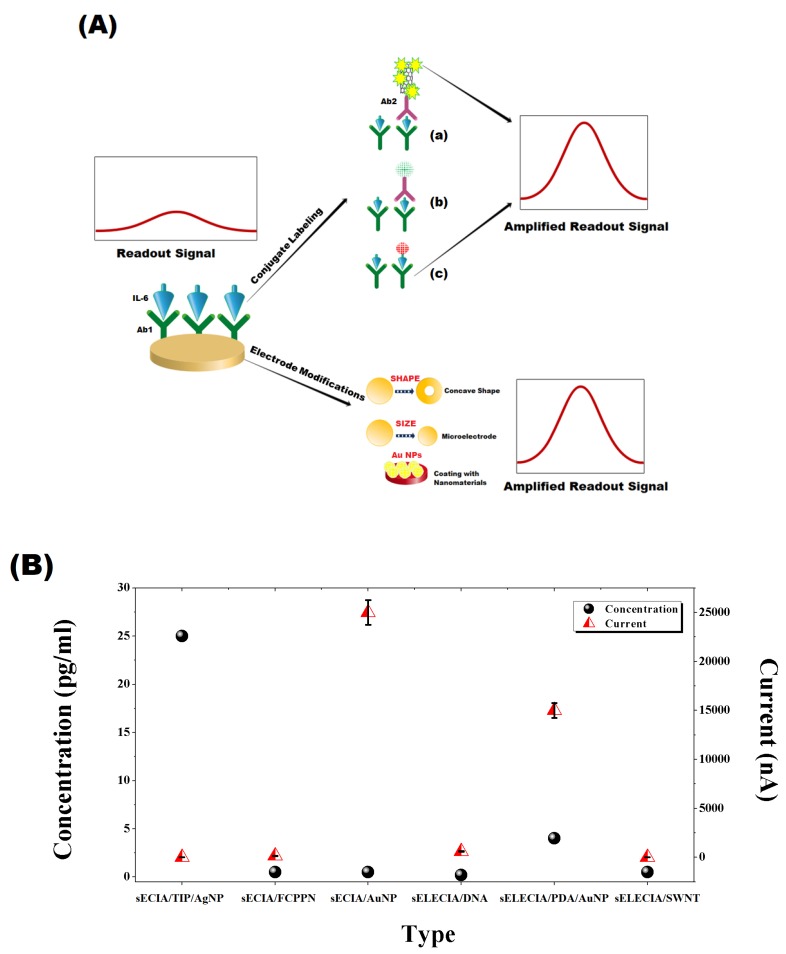
(**A**) Signal amplification strategies: (**a**) use of combination of nanomaterials and multi-enzyme labels on a secondary antibody to enhance the outgoing signal, (**b**) use of electro-active (AuNPs or electro-conductive polymeric) NPs, (**c**) a competitive approach in which analyte labeled with electro-active nanoparticles competes towards complex formation with Ab${}_{1}$ immobilized on electrode surface. Electrode modification approach involves controlling geometry and coating with conductive nanoparticles to boost the change in detecting signal. (**B**) Signal to Least detectable concentration plot for different ECIA scheme sECIA/Titanium Phosphate (TiP)/AgNP [105]; sECIA FC/PPN [107]; sECIA/AuNP [106]; sELECIA/DNA [110]; sELECIA/PDA/AuNP [109]; sELECIA/SWNT (single-walled carbon nanotubes) [108].

The geometry of the electrode’s surface plays a pivotal role in preventing the counter-ions screening at the double layer [123], and a concave surface promotes efficient electron transfer kinetics, thereby increasing the sensitivity of the sensor. An array of eight gold disc-shaped microelectrodes was engraved on a silicon needle with an objective of real-time in-situ monitoring of IL-6 [100] (Figure 4A). This approach was envisioned following the development of a robust microelectrode-based electrochemical sensing platform for chemical and DNA sensing applications [124,125,126,127]. Figure 3a shows the equivalent circuit formed at the electrode-electrolyte interface. The interfacial chemistry at the surface of electrode can be best explored by electrochemical impedance spectroscopy due to the associated sensitivity [127,128,129,130,131]. The capture antibody, immobilized through an organic linker at the Au disc electrode’s surface, permits detection by increasing charge transfer resistance (R${}_{ct}$). Contrary to the macro-electrode’s direct relation with R${}_{ct}$ [126,132], it was found to decrease due to an increase in effective surface area, and the linear response of the current was consistent with the results reported in [130,133,134]. In this study, the contribution of nonlinear resistance (R${}_{nl}$) due to a steady current represented by R${}_{nl}$ was also decreased to 14%. It was a simple method to augment sensitivity and improve linear response of the sensor compared to the ones that require nano-labels [135].

Nanolabeling of the detecting antibody or modification of the electrode surface with conductive nanomaterials offers a fascinating alternative approach to improve a sensor’s performance. Figure 3b shows the structure developed by Tertis et al. [101], where a faster electron transfer kinetics was achieved by improving the immobilization efficiency of IL-6 specific aptamer through electrochemical deposition of polypyrrole and gold nanoparticles on a screen-printed graphite electrode. Yang et al. [103], in 2013, used SWCNT with AuNPS to immobilize anti-IL-6 on silicon substrate, and its equivalent circuit is shown in Figure 3c. Inclusion of SWCNT/AuNP in a sensor’s structure adds an additional resistor (R${}_{1}$) and a constant phase element (CPE${}_{1}$) [103], whereas R${}_{2}$ and CPE${}_{2}$ are charge transfer resistance and double layer capacitance of bio-recognition layer, respectively. We have also included an example of a sandwich-based enzyme linked electrochemical assay (sELECIA) [109] as a model of complex interfaces formed by the bio-recognition layer with thickness more than 1 Debye. The use of CNT provides sufficient surface area to carry 5/6 labels of enzymes per conjugate antibody. The presence of AuNP paves the way for electrons rushing towards an electrode, produced by the catalytic events upon substrate addition [136]. The introduction of a conjugate antibody labeled with an enzyme and nanolabel will form an interface that has an equivalent circuit with a three-serial combination of parallel-connected resistors and constant phase elements (Figure 3d).

### 2.3. Signal Amplification

Different schemes of sensing layer, as discussed earlier, were proposed with an aim to amplify the readout signal which ultimately improves sensitivity and dynamic range of biosensor. Some of the effective approaches which significantly boost the outgoing signals of sensor are (1) modification of electrode geometry and size [100]; (2) coating of electrode with nano-features, such as SWCNT, AuNP, and Polypyrrole nanoparticles [101,102,103,104]; and (3) labeling of conjugate in sandwich configuration with nanoparticles, enzymes or a combination of two [105,106,108,109,110] (Figure 4A). Figure 4B shows a comparison of intensity of readout signal against the least concentration detected by each configuration, i.e., direct and sandwich electrochemical immunoassay. It is evident that tailoring electrode’s size and geometry did not amplify signal as effectively as coating it with gold nanoparticles in sandwich, as well as direct ECIA. The amount of signal produced against the limit of detection is also very high, which also explains its sensitivity and wide linear response. Titanium phosphate (TiP) nanospheres loaded with silver nanoparticles also greatly enhance the sensitivity and showed a wider dynamic range. In contrast, dual signal amplification strategy with DNA probes and carbon nanotubes provide a moderate increase in current response against the least detectable concentration.

### 2.4. Sensitivity and Selectivity

**Figure 5:**
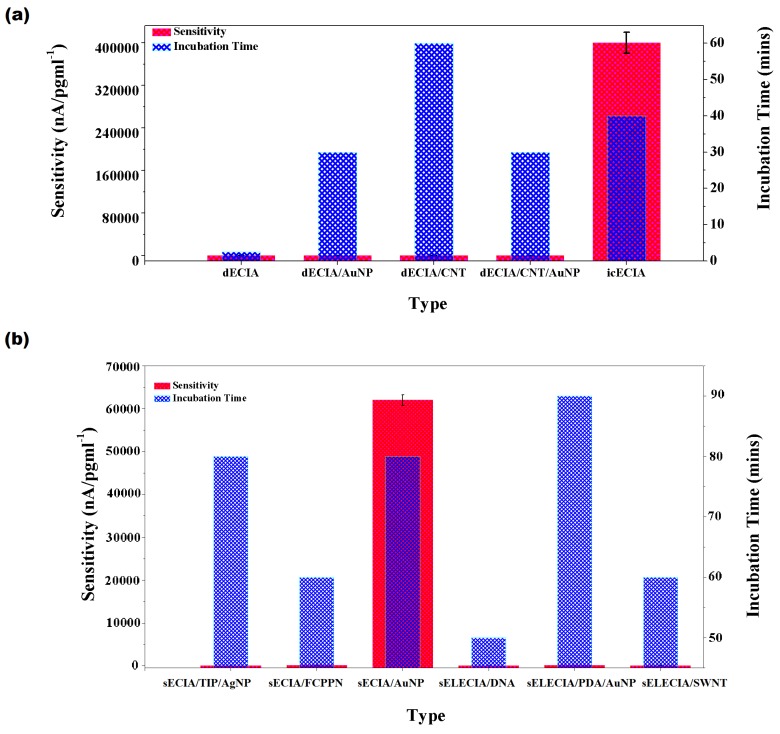
Sensitivity of (**a**) direct electrochemical immunoassay/dECIA and indirect competitive electrochemical immunoassay/icECIA (calculated/obtained from [100,101,102,103,104]); (**b**) sandwich electrochemical immunossay/sECIA with nanomaterial or enzyme label/sELECIA (calculated/ obtained from [106,107,108,109,110,137]).

We calculated the sensitivity of the biosensors and compared it, along with respective incubation times, in Figure 5. Sensitivity is dependent on two important factors: immobilization efficiency of detecting antibody and double layer screening. It should be recalled that the double layer screening effect becomes pronounced in sECIA/NP and sELECIA due to the additional conjugate antibody and label. Interestingly, the greatest sensitivity was exhibited by sandwich ECIA, which utilized AuNPs as an electro-active label for conjugate and upon binding with target analyte, it was used as a seed to enlarge the size of bound AuNP in presence of equimolar HAuCl${}_{4}$ and ascorbic acid, while cetyl trimethyl ammonium bromide(CTAB) was included in the solution as a surfactant [106]. It is believed that CATB/AuNP mediated electron transfer at interface by providing increased surface area and electrostatic attraction to the redox probe, which resulted in improved current response at the electrode. The indirect competitive ECIA introduced by Lou et al. [104] (2014) also showed a comparable sensitivity with less incubation time. He used electrode which was coated with Au and PdNPs. For competitive sensing, he used IL-6 immobilized on dopamine-coated polystyrene spheres that were decorated with silver nanoparticles/AgNP. The high signal-to-noise ratio was achieved due to synergistic effect of Au/PdNPs, AgNP and heated carbon electron technique resulting in unimpeded electron transfer kinetics. Electrode modification or conjugate labeling with single-wall or multiwall carbon nanotube adds up resistive effect in amperometric technique, its presence produces a negative effect on the readout signal. It can be hypothesized that unexpectedly low sensitivity associated with remaining direct or sandwich-based assay was due to the unfit combination of detecting signal and the nanomaterial used for signal amplification purpose. A liquid-gated field effect transistor-based biosensor for IL-6 detection was developed by Chen et al. [102] (2016). The source and drain electrodes were made of gold, and SWCNT was used to immobilize the primary detecting antibody. He observed a current measuring 4 nA in response to the lowest analyte concentration, i.e., 1.37 pg/mL. In a recent study, Khosravi et al. [111] (2017) used an aptasensor immobilized atop a CN-based FET sensor through a 1-Pyrenebutanoic Acid Succinimidyl Ester (PASE) linker. To deal with the counter-ions screening effect, which limits the applicability of FET-based sensing technology, aptamers were used instead of antibodies in the CNT FET sensor [111]. Interestingly, the sensor’s performance in blood samples was found similar to that of LGFET introduced by [102] (Table 2). On the contrary, if we compare the latter approach with the aptasensor introduced by [101], we notice it exhibits the greatest sensitivity with a wider dynamic range.

Specificity is another important attribute as non-specific binding (NSB) at the surface produces a false signal and gives misleading results. Human blood and serum is a complex mixture of proteins, and they share structural similarities; therefore, the sensor’s construct should smartly recognize its target and quantitatively analyze its concentration in the given sample. We looked for the established electrochemical biosensor for IL-6 detection that was tested under proper conditions to assess its affinity for NSB. The icECIA developed by Lou et al. [104] (2014) showed specific binding to IL-6 in ten-fold higher concentrations of bovine serum albumin (BSA), matrix metalloproteinases 2 (MMP-2), and carcinoembroyinc antigen (CEA). LG-FET for IL-6 detection was tested for NSB with equal concentrations of IL-6, BSA, and Cysteine A, and the drain current produced by specific binding of analyte was 18.7%, whereas a <3% false signal was generated by NSB [102]. Sandwich ECIA with CTAB-stabilized AuNP as electroactive label also gave specific a response to IL-6 in ten-fold higher concentration of IgG, BSA, α-fetoprotein, and lysozyme [106]. All the sandwich ECIA structures were found to have higher specificity to analyte because the readout signal is produced after two successive antigen–antibody complex forming events. The conjugate antibody with electroactive or enzyme label assures the antigen-specific response of the biosensor [105,106,107,109,110].

An alternate effective approach to prevent NSB is the careful selection of blocking agent or anti-fouling coating because a false positive signal is an outcome of unreacted sites at electrode’s surface and complex proteins present in plasma or serum samples [25,101,103,104,138]. A widely-used blocking buffer is Bovine Serum Albumin (BSA) in combination with a porous polymeric network [103,104,138]. Electrodes coated with BSA after immobilization of detecting antibody produce a highly specific response with 4 to 10% RSD (root mean square deviation) in the presence of 10–100-fold higher concentration of non-specific proteins [103,104,138]. In contrast, Tertis et al. [101] used 6-mercapto-1-hexanol (MCH) to stabilize bound aptamers and block unreacted sites at the surface of electrochemical aptasensor designed for real-time monitoring of IL-6. Specificity testing was performed in a mixture of interfering proteins at 100-fold higher concentration, and they attained 96.74% recovery results with an RSD of 0.64% [101].

### 2.5. Dynamic Range

As discussed earlier, the level of IL-6 in the human body is <6 pg/mL under normal conditions. It increases considerably in many pathological conditions, depending on type of disease and severity; for instance, in a case of chronic sepsis, it goes up to 100,000 pg/mL [36]. The range within which a biosensor demonstrates a linear response between readout signal and concentration of detecting analyte is referred as dynamic range. While designing sensor’s scheme, it is crucial to consider the factors that affect the resulting dynamic range, such as immobilization efficiency of the primary antibody and the effective electrode surface area that is accessible to analyte for binding and faster electron transfer kinetic. The widest linear response with limit of detection (LOD) 0.33 pg/mL was observed in dECIA with a Au/PPyNP-coated electrode [101]. The highest concentration that it can detect is 15,000,000 pg/mL. This implies that the electroactive label used exhibits a synergistic effect with the IL-6-specific aptamer on the sensitive and linear response. The dynamic range of indirect competitive ECIA was 0.1–100,000 pg/mL [104] (Table 2). The competitive nature of the assay has reduced the LOD to 0.059 pg/mL, and the immobilization efficiency offered by AuNP provides a good dynamic range. Carbon nanotubes (CNs), when used as the label in both sandwich and direct ECIA, result in a narrow dynamic range despite the high stability it offers, and this makes it an unattractive approach for the development of a facile amperometric-based electrochemical sensor [103,108,109].

### 2.6. Stability

We observed longest shelf life of LGFET with 20% degradation in sensitivity after three months [102]. The intact sensing layer remained stable this long due to the strong adhesive properties of CNT. An electrode coated with AuNP for icECIA gave a 90% initial response when tested after storing it at 4 °C for 14 days [104]. Yang et al. [103] (2013) observed a similar initial response of a fabricated electrode after one month of storage at 4 °C. The second longest shelf-life was observed in sECIA developed by Peng et al. [105] (2011), and the sensor showed 94.2% of the initial response after two months. It is primarily due to the stability of AgNP-decorated TiP nanospheres, which showed less than 6% deterioration in its signal amplification efficiency. Dual-amplification strategy was first introduced by Wang et al. [109] in 2011, and they used CNT to provide increased surface area for attachment of multiple HRP labels. He successfully increased the number of HRP labels for single conjugation event. The capture antibody was immobilized on sensing electrode through Au-S linkage. When the immunosensor was tested after thirty days, it showed 85.5% of the initial response. We have noticed a consistent increase in the stability of the biosensor with the use of AuNP and CNT as label or linker to immobilize the primary antibody. We can deduce that stability of the immunosensor is dependent on the affinity and strength of the bond that a label forms with biomolecules.

## 3. Optical Biosensor

An extensively studied and well-established method for the quantitative detection of IL-6 is optical sensing. It is based on the light scattering properties of the biomolecules to study bound antigen-antibody complexes. The variants of optical biosensors that have been introduced so far differ in coupling agent, conjugate label, detecting signal, and transduction model to read the sensor’s output [137,138,139,140,141,142,143]. The following sections discuss the general architecture of optical biosensors designed for sensitive detection of IL-6 and the signal amplification strategies adopted in each category to enhance the overall performance of the biosensor.

### 3.1. Sensor Structure

We will briefly discuss the structure of optical biosensor because the components of the sensing layer are nearly same in both categories except the transduction model and conjugate labels, which are commonly fluorophores for optical sensing. A widely used method to prevent non-specific adsorption at the sensing layer is sandwich immunoassay because it utilizes a conjugate system, which generates a signal corresponding to the analyte concentration [137,139,140,141,142,143]. Interestingly, we found a novel indirect competitive photo-electrochemical method, which we call hybrid method [138]. It is based on co-sensitization strategy, and the sensor surface is coated with a semiconductor layer. The competitor is also labeled with a semi-conductor nanomaterial, which upon binding with the captured antibody, produces a photocurrent when excited with white light with a spectral range of 200–2500 nm. The photocurrent produced by the sensor’s surface has an inverse relation with a concentration of IL-6. We calculated the sensitivity from the calibration curve provided in [104] and found it at 3340 nA/pg mL${}^{-1}$, while the dynamic range it offers is 1–100,000 pg/mL with LOD 0.38 pg/mL. We noticed that the linear response it generated is similar to the one we observed in the indirect competitive electrochemical immunoassay [104].

### 3.2. Signal Amplification Strategies in Optical Biosensor

Herein, we will elaborate the impact that chosen signal amplification strategy has on the sensor performance. The output signal can be greatly enhanced by a careful selection of coupling agent, conjugate label, detecting signal, and transducer. We will discuss few examples from each of the mentioned approaches in the subsequent sections.

#### 3.2.1. Coupling Agent

The preparation of selective self-assembled monolayer (SAM) on the glass substrate, which blocks NSB and offers higher immobilization efficiency of the captured antibody, is a major challenge, while the latter property is heavily dependent on the choice of coupling agent. A highly adhesive SAM ensures sensor stability [144] and permits sensitive detection of analyte at higher concentrations. Different coupling strategies have been adopted to find out its effect on the outgoing signal.

**Figure 6:**
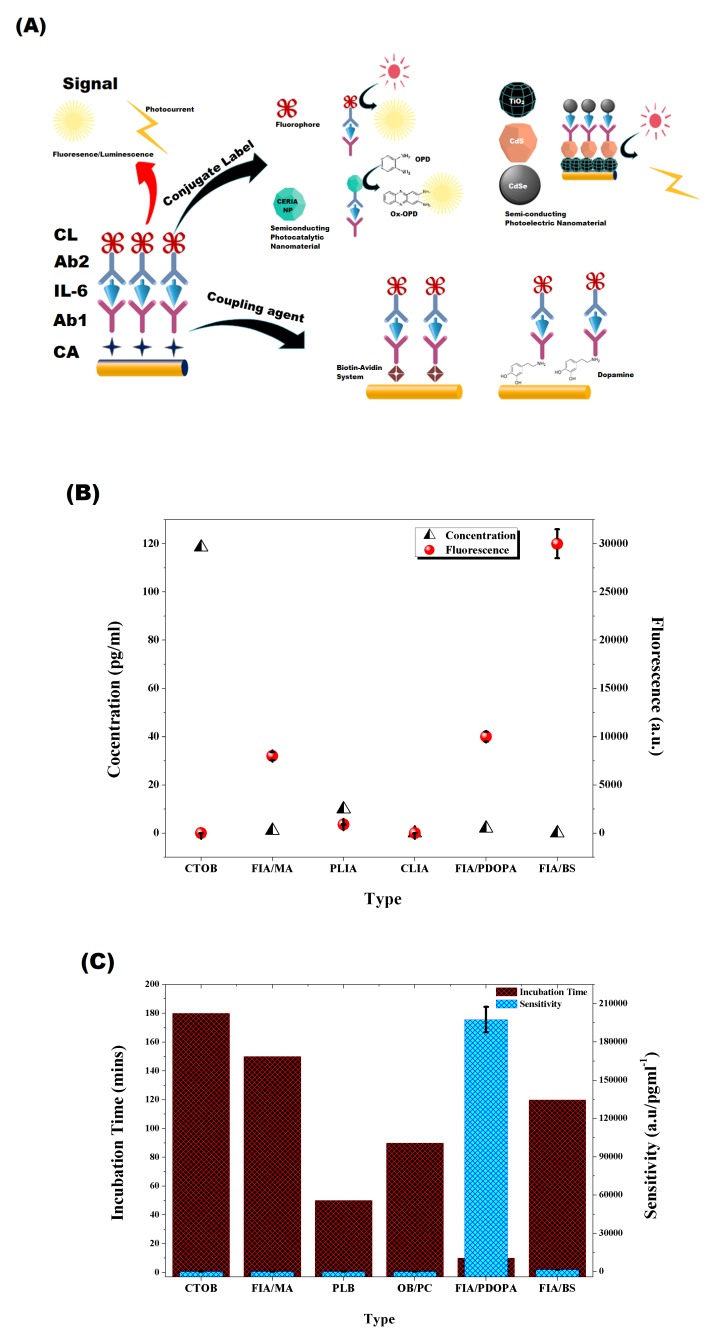
(**A**) Signal amplification strategies adopted in optical biosensors for IL-6 detection. (**B**) Signal to concentration plot for different optical biosensors (calculated/obtained from [137,139,140,141,142,143]). (**C**) Sensitivity of optical biosensors (calculated/obtained from [137,139,140,141,142,143].)

Toma and Tawa [140], in 2016, chose polydopamine (PDA) as the coupling agent for a surface plasmon-enhanced fluorescence spectroscopy (SPFS)-based sensor for IL-6 detection (Figure 6A). It has already been used as bifunctional linker in many biosensing platforms developed for diverse application and was found to enhance the SPFS signal [145,146,147,148]. The thickness of the PDA layer on glass substrate coated with gold was optimized to 1.5 nm to achieve the best SPFS response. The detecting/secondary antibody was labeled with Alex Fluor 647 and produced fluorescence following the reaction with IL-6-Anti IL-6 complex. The inevitable change in PDA-associated sensitivity and increase in dynamic range for the fluorescence immunoassay (FIA) diversifies its application in clinical diagnosis with minimum incubation time (Figure 6C and Table 3). In contrast, Zang et al. [139] developed a biotin–streptavidin system (BAS) in 2018 to couple biomolecules on the sensor’s surface (Figure 6A). The strong covalent interaction of the biotin–streptavidin complex [149,150] helps it sustain extreme chemical conditions [151] and makes it a favorable candidate for surface modification applications [152,153]. BAS involved attachment of sulfo-NHS-Biotin on a glass substrate, which was further modified with the streptavidin molecule with multiple active sites facing outward. Biotinylated-antibody for IL-6 was immobilized on the surface, and superparamagnetic oxide conjugated secondary antibody was used for fluorescence-based detection. Surprisingly, the highest signal-to-LOD ratio was obtained with the BAS coupling approach, but it generated a narrow linear response, which limits its prognostic capabilities required for clinical applications (Figure 6 and Table 3). Its sensitivity remains comparable to other FIA-based IL-6 detection methods (Figure 6B).

#### 3.2.2. Conjugate Labeling

The sensitivity and dynamic range of optical sensors is dependent on the type of photoemissive material used to label conjugate antibody. Generally, Fluorophore is used for labeling, but photobleaching and reduced photostability are the common issues which appear in the detection methods based on FIA (Figure 6A). Fluorophores are organic dyes which are attached with secondary/ conjugate antibody and produce characteristic fluorescence from Near IR to visible range. Alexa Fluor, Dy647, and fluorescein isothiocyanate are widely used examples in sandwich fluorescence immunoassay [140,142,143]. Table 3 shows a comparison of optical biosensors for IL-6 detection with a combination of different signal amplification strategies. It is worth noting that, despite of the fact that FIA is a well-established and commercially used method of quantitative analysis, it offers narrow dynamic range and high detection limit compared to others. Figure 6B demonstrates the signal to least detectable concentration (LOD) plot, and it can be deduced from the improved signal to LOD ratio that, despite the narrow dynamic range, it accurately determines the concentration of analyte as the output signal is significantly enhanced.

An alternative approach to facile optical detection is the use of semi-conducting nanomaterials that exhibit photocatalytic [137], photoelectric [138], or photoluminescence [141] properties for conjugate labeling. Ceria nanospheres exhibit photocatalytic activity [154,155,156,157,158] against organic dye [159,160], and it was used to label anti-IL-6 by [137] (Figure 6A). A calorimetric response was generated by a Ceria-labeled conjugate antibody following oxidation of *o-phenylenediamine* which was used as a substrate (Figure 6A). The developed chemiluminescence immunoassay (CLIA) can detect interleukin-6 up to 10,000 pg/mL with a reasonable sensitivity; however, the signal to LOD ratio is not sufficiently enhanced, which compromises the accuracy of the sensor (Figure 6B,C). Semiconductor-based quantum dots with near infrared photoluminescence properties have gained popularity in bio-imaging [161,162,163] and biosensing due to its photostable nature. Quantum dots with less than 5.5 nm diameter have less autofluorescence-related noise [164]. Based on these observations, Xiong et al. [141] in 2013 introduced copper indium sulfide and zinc sulfide (CuInS${}_{2}$/ZnS)-based Photoluminescence Immunoassay (PLIA). Photoluminescence (PL) from CuInS${}_{2}$ nanocrystal is due to the donor-acceptor interaction and an optimized Cu-to-In ratio not only increases the intensity of photoluminescence but also its duration to 690 ns [165]. The spectral range can be tailored from Visible to Near infrared (NIR) range (540–680) by synthesizing the core-shell structure of CuInS${}_{2}$/ZnS [166]. The diameter of quantum dot was 3.3 nm, used to label conjugate antibody against IL-6. Characteristic PL spectra was obtained when analyzed through fluorescence spectrophotometer after the conjugation reaction. PL intensity showed direct relation with IL-6 concentration. LOD with PLIA reduced to 0.04 pg/mL, and the dynamic range increased to 20,000 pg/mL [141].

A dual co-sensitized strategy was implemented by Fan et al. [138] (2014) for signal amplification of semi-conductor-based optical biosensors. He coupled a semiconductor with smaller and larger band gaps to improve light absorption properties of the semiconductor nanoparticles coated on an Indium Titanium oxide (ITO) electrode. The sensor architecture was designed for indirect competitive photoelectrochemical (icPECIA) detection of IL-6, and the primary antibody was immobilized on cadmium sulfide (CdS)-coated substrate through cross-linking with chitosan. IL-6 was conjugated with cadmium selenide (CdSe) nanoparticles and used as a competitor (Figure 6A). An optimized thickness of CdS was used to maximize light absorption [167] and to prevent R${}_{s}$-associated signal degradation [168]. The stepwise band edge gap structure produced an ultra-sensitive sensor (3340 nA/pg${}^{-1}$) due to faster transfer kinetics of photogenerated electrons [169]. The high sensitivity in icPECIA can be attributed to the photocurrent conversion efficiency of the co-sensitized construct. The linear response of the calibration curve was also extended to 100,000 pg/mL (Table 3).

#### 3.2.3. Detecting Signal

The nature of the input signal used for quantitative detection requires special attention because the sensing architecture and signal amplification are based on it. This ultimately defines the accuracy and reliability of output signals. In the case of optical biosensors designed specifically for the detection of IL-6, we witnessed the use of different forms of light, such as that from visible range [137], Near IR [141], fluorescence [139,140,142,143], and photocurrent [138]. Different approaches were introduced to enhance the fluorescence intensity in response to minimum detectable concentration, like decorating conjugate with multiple fluorescent molecules [143] and use of combination tapered optic fiber [142]. A dual amplification strategy was devised by Toma and Tawa [140] (2016), and they used PDA as the coupling agent in combination with surface plasmon-enhanced fluorescence spectroscopy (SPFS), and they attained a tremendous improvement in sensitivity with minimum incubation time. The coupling of SPFS in other intended biosensing applications report a three-fold increase in sensitivity [170,171,172,173]. However, the CLIA scheme proposed by Peng et al. [137] (2016) exhibits wider dynamic range.

Fan et al. [138] (2014) used light as an excitation source on photoactive semi-conducting material to develop a PECIA for IL-6 detection (Figure 6A). The optical readout signal has the limitation due to undesirable noise that comes with autofluorescence and the photobleaching effect, which is responsible for signal deterioration. To counter these drawbacks, exploiting photoelectrochemical response for quantitative analysis is a more fascinating approach (Figure 6A). It offers a facile, integrable, and ultrasensitive platform, more specifically when used to design indirect competitive electrochemical immunoassay. In addition to this, it provides procedural simplicity with shorter incubation time. The competitive advantage of this method over CLIA is that dynamic range has increased tend-fold (Table 3).

Single Molecular Array/SiMoA Technology was introduced by Rissin et al. [174] in 2010 which upgraded ELISA and allowed detection of proteins in a digitized manner. A single enzyme catalyzed event can be detected by introducing conjugated complex in a femtoliter sized microarray of 50,000 wells. The wells are designed to hold a single magnetic bead, and substrate is introduced after dispensing conjugated complex in the micro-arrays. This technique was initially designed to detect tumor markers which are present at sub-picomolar concentrations in the blood, and it was later utilized by [175] to detect TNF and IL-6 in plasma of Crohn’s disease patients. A comparison of the SiMoA-based FIA in Table 3 makes it evident that it offers thousand-fold greater sensitivity compared to conventional ELISA; however, its dynamic range is limited to 250 pg/mL.

## 4. Conclusions

IL-6 has pivotal role in manifestation of many diseases, including cancer and sepsis. Its level in patient’s serum can facilitate timely diagnosis and treatment. The prognostic level of IL-6 varies from 5 to 100,000 pg/mL, depending on the nature and severity of a disease. Many variants of optical and electrochemical biosensors have been introduced during the past decade, with an aim to combine all desirable properties of the ideal sensor, i.e., ultra-sensitivity, assay simplicity, integrability, and reliability. One important factor to consider here is that developed sensor architecture should exhibit dynamic range which falls within the prognostic levels of IL-6. Fluorescence immunoassay (FIA) is a well-established and commercially-used method for detection, but it has the limitation of narrow dynamic range. The attempts to widen the detection range while maintaining sensitivity have led to the development of SPFS platforms which offer a three-fold increment in sensitivity, with shorter incubation time. The sandwich-based configuration exhibits longer incubation duration due to complex multi-step reactions. Signal amplification approaches that include careful selection of coupling agent and attachment of multiple fluorescent labels with conjugate provided a facile detection method with considerable improvement in sensitivity; however, the maximum increase in dynamic range was found at 11,859 pg/mL.

Photobleaching of the fluorophores that are used to label conjugate compromises the reliability of optical signal. The decrease in signal intensity produces a false signal and generates misleading quantitative analysis. Electrochemical biosensors were introduced to counter this effect and include integrability features. We can confidently deduce, after critically analyzing the performance of ECIA-based detection methods, that it holds immense potential for commercial applications because it offers real-time, in-situ monitoring with a dynamic range up to 100,000 pg/mL and LOD in fg/mL. The incubation time is also reduced, and the most sensitive indirect competitive approach permits single-step detection of analyte. In ECIA methods, we have noticed that shelf life of biosensors increases when the conjugate label used is either AuNP or CNT. On the contrary, CNT augments charge transfer resistance in amperometric detection technique, thus limiting its applicability. A single-step indirect competitive assay was found to be a favorable approach in developing a high fidelity biosensor. A comparison of icECIA and icPECIA shows that the latter is a more cost-effective method, with the same dynamic range. The charge separation properties of icPECIA were excellent because of the difference in excitation and emission source. In addition, photogenerated electrons have ultrafast transfer kinetics when the co-sensitization strategy is used for signal amplification. We suggest that icPECIA-based biosensors provide a technically feasible and facile detection method and have potential for commercialization.

## Figures and Tables

**Table 1:** Level of IL-6 in different pathological conditions.

Disease	Interleukin 6 Level (pg/mL)	Reference
Alzheimer’s Disease	85–567	[41]
Meningitis	450–32,000	[42]
Myocardial Infarction	28.5–46.5	[43]
Cardiac Myxoma	greater than 56	[44]
Multiple Myeloma	5–33	[45]
Burkitt Lymphoma	100.3	[46]
Post-Transplant Lymphoproliferative Disease/PTLD	143–11,020	[47]
Cachexia	100	[48]
Rheumatoid Arthritis	17	[49]
Psoriasis	30,000	[50]
Hepatitis B Virus Infection/Hepatocyte Carcinoma	7–18.9	[51]
Sepsis	5000–100,000	[36]

**Table 2:** Properties of different types of electrochemical immunoassay-based sensors for IL-6 detection. SWNT = single-walled carbon nanotubes; AuNPs = gold nanoparticles.

Detection Method	Technique	Receptor	Analyte & Conjugate	Signal Type	LOD ^†^	Dynamic Range ^†^	Ref
Direct ECIA	CV, EIS, DPV	Anti-IL-6/Gold ME	IL-6	R${}_{ct}$ and Current	20	20–100	[100]
Direct EC aptasensor assay	CV, EIS	PPyNS/AuNP/IL-6-Aptamer	IL-6	R${}_{ct}$	0.33	1–15,000,000	[101]
Direct EC aptasensor	FET	CNT/PASE/IL-6-Aptamer	IL-6	Drain Current	1	1–100	[111]
Direct ECIA	LGFET	CNTFET/Anti IL-6	IL-6	Drain Current	1.37	1–100	[102]
Indirect competitive ECIA	EIS, LSV	ERGO/AuPdNP/Anti IL-6	PS/PDA/AgNP/IL-6	R${}_{ct}$ and Current	0.059	0.1–100,000	[104]
Direct ECIA	EIS	CNT/AuNP/Anti IL-6	IL-6	R${}_{ct}$ t	0.00001	0.00001–0.1	[103]
Sandwich ECIA/NP	CV, DPV	SPION/Ab${}_{1}$	TiP/AgNP/ Ab${}_{2}$/IL-6	Current	0.1	0.00001–10,000	[105]
Sandwich ECIA/NP	CV, SWV	GC/GO/Ab${}_{1}$	FC/PPN/ Ab${}_{2}$/IL-6	Current	1	2–20,000	[107]
Sandwich ECIA/NP	CV,(SWV)	11-MUA/Ab${}_{1}$	CTAB(AuNP)/ Ab${}_{2}$/IL-6	Current	2	5–50,000	[106]
Sandwich ELECIA	Amperometry	GCE/MWCNT/Ab${}_{1}$	S1-Avidin/Biotin-Ab${}_{1}$ (+) S2-HRP-S3(TargetDNA)	Current	0.05	0.2–20	[110]
Sandwich ELECIA	EIS, Amperometry	ITO/PDA/AuNPs/Ab${}_{1}$	CNT/PDA/AuNP/ Ab${}_{2}$-HRP-IL-6	Current	1	4–800	[109]
Sandwich ELECIA	Rotating Disc Amperometry	SWNTForest/Ab${}_{1}$	Ab${}_{2}$-Biotin/(m)HRP-Streptavidin/(h) Ab${}_{2}$-MWNT-HRP	Current/Absorbance	0.5	0.5–30	[108]

^†^ mentioned in pg/mL.

**Table 3:** Properties of optical biosensors designed for IL-6 detection.

Detection Method	Technique	Receptor	Conjugate	Signal Type	LOD ^†^	Dynamic Range ^†^	Ref
FIA ^⋄^	Fluorescent Imaging	OpticFiber/Streptavidin-Biotin/Ab${}_{1}$	Ab${}_{2}$/FMNPs	Fluorescence	0.1	0.4–400	[139]
FIA ^⋄^	SPFS ^γ^	Glass/AuNP/PDOPA/Ab${}_{1}$	Ab${}_{2}$/AlexaFluor647	Fluorescence	2	2–2372	[140]
FIA ^⋄^	FIA ^·^	Ab${}_{1}$/IL-6	Dy647/Streptavidin/MNP/Biotin-Ab_2_	Fluorescence	20	1.1–1000	[143]
CLIA ^▽^	UV-Vis Spectroscopy	Ab_1_/Fe_3_O_4_MNP	Ab_2_/CeO_2_NP	Absorbance	0.04	0.1–10,000	[137]
Indirect Competitive PECIA ^‡^	EIS, Amperometry	Ab_1_/CS/CdS//TiO_2_/ITO	IL-6/CdSe	R${}_{ct}$ & I${}_{p}$	0.38	1–100,000	[138]
PLIA ^☆^	Photoluminescence	PDA/PDMS/AuNP/Ab${}_{1}$	Ab_2_/CuInS_2_/ZnSNCs	Fluorescence	0.02	20–20,000	[141]
CTOB ^∓^	FIA ^·^	CTOB/APTS/Sulfo-SMCC/Ab${}_{1}$	Alex488/Ab${}_{2}$/ and IL-6	Fluorescence	120	118–11,859	[176]
SiMoA ^♣^	Digital ELISA	Micro Magnetic Bead/Ab${}_{1}$/IL-6	Biotin-Ab${}_{2}$/SβG ^*δ*^	Fluorescence	0.006	0.006–250	[175].

^†^ mentioned in pg/mL. ^⋄^ Fluorescence Immunoassay. ^☆^ Photoluminescence Immunoassay. ^▽^ Chemiluminescence Immunoassay. ^∓^ Combination Tapered Optic Biosensor. ^‡^ Photoelectrochemical Immunoassay. ^γ^ Surface Plasmon Enhanced Fluorescence Spectroscopy. ^·^ Fluorescence Imaging Analysis. ^♣^ Single Moleculare Array. ^δ^ Streptavidin-β-Galactosidase.

## Abbreviations

The following abbreviations are used in this manuscript:

IL-6	Interleukin 6
IL-6R	Interleukin 6 Receptor
TNF	Tumor Necrosis Factor
ACTH	Adrenocorticotrophic Hormone
CRF	Corticotrophin Releasing Factor
GCC	Glucocorticoid Hormone
CRP	c-reactive Protein
AD	Alzheimer’s Disease
LPL	Lipoprotein Lipase
dECIA	Direct Electrochemical Immunoassay
icECIA	Indirect Competitive Electrochemical Immunoassay
sECIA-NP	Sandwich Nanoparticles Labeled Electrochemical Immunoassay
sELECIA	Sandwich Enzyme Linked Electrochemical Immunoassay
FIA	Fluorescent Immunoassay
CLIA	Chemiluminescent Immunoassay
PECIA	Photo-Electrochemical Immunoassay
PLIA	Photoluminescence Immunoassay
PPy	Polypyrrole
CNT	Carbon Nanotubes
CTOB	Combination Tapered Optic Fiber Biosensor
